# Large Accumulation of Collagen and Increased Activation of Mast Cells in Hearts of Mice with Hyperlipidemia

**DOI:** 10.5935/abc.20170143

**Published:** 2017-11

**Authors:** Yunpeng Cheng, Yanqiu Zhu, Jiashu Zhang, Xingping Duan, Ying Zhang

**Affiliations:** 1 Department of Cardiology - the First Affiliated Hospital of Dalian Medical University – China; 2 Department of Ultrasonics - Affiliated Zhongshan Hospital of Dalian University – China; 3 College of Pharmacy - Dalian Medical University - China

**Keywords:** Rats, Hyperlipidemias / physiopathology, Heart, Fibrosis, Leukemia, Mast-Cell

## Abstract

**Background:**

Hyperlipidemia, which is characterized by an elevation of lipids in the bloodstream, is a major risk factor for cardiac disease.

**Objectives:**

The present study investigated the role of fibrosis in the progression of hyperlipidemia in the mice heart, and whether mast cell activation was associated with the fibrosis process.

**Methods:**

Hyperlipidemia was produced in C57BL / 6 mice by feeding them on a high-fat diet for 8 weeks.To assess tissue fibrosis, picrosirius red staining was performed. Hematoxylin & eosin (H&E) staining was performed to identify the histopathological changes in the hearts. Immunohistochemistry was also accomplished to determine the localization of transforming growth factor (TGF)-β and α-smooth muscle actin (α-SMA). Western blotting was performed to analyze the expression of chymase, tryptase, TGF-β, α-SMA and activity of Wnt/β-catenin pathway. At the end, serum total cholesterol (TC) and triglycerides (TG) levels were measured. All the values were expressed as means ± SD, the statistical significance level adopted was 5%.

**Results:**

Hyperlipidemia mice showed significantly increased collagen deposition in the hearts compared with normal mice. In addition, H&E staining showed significant cellular degeneration. Cardiac muscle was arranged in disorder with fracture in mice of the model group. Immunohistochemistry and western blot analysis revealed that expression levels of tryptase, chymase, β-catenin, TGF-β and α-SMA were significantly increased in the hyperlipidemia mice compared with the control group.

**Conclusions:**

The results indicated that mast cell activation might induce cardiac fibrosis by tryptase and chymase in hyperlipidemia, which had a close relationship with the increased activity of TGF-β/Wnt/β-catenin pathway.

## Introduction

Hyperlipidemia refers to hypercholesterolemia, hypertriglyceridemia and mixed hyperlipidemia, and is a very common biochemical disorder^1^ with significantly risk of cardiovascular disease.^2,3^ It is reported that the hyperglycemia, another metabolic disorder, has a strong association with cardiac fibrosis that may reduce myocardial compliance and contribute to the pathogenesis of heart failure.^4,5^ However, the role of the hyperlipidemia in the development of cardiac fibrosis is poorly understood.

Cardiac fibrosis, which is a common pathologic feature of end-stage heart disease, always results in serious cardiac dysfunction.^6,7^ Therefore, potential causes of cardiac fibrosis have to be investigated. Although cardiac fibrosis is an important stage in the progression of heart disease, the mechanisms underlying fibrosis development and progression are still unclear. The process of fibrosis is mechanically characterized by myofibroblast accumulation, collagen deposition, extracellular matrix remodeling, and increased tissue stiffness, so that impairs the organ's function by reducing tissue elasticity and compliance. As reported, the Wnt/β-catenin/TGF-β signaling pathway is a key mediator of fibroblast activation^8,9^ and play important role in driving the aberrant synthesis of the extracellular matrix in heart fibrotic diseases.^10,11^

Mast cells have been recognized as important effector cells in tissue fibrosis.^12,13^ Potentially mast cell activation and degranulation could result in the release of inflammatory and profibrotic mediators promoting tissue fibrosis.^14^ Mast cells express serine proteases: tryptase and chymase, are associated with fibrosis in various diseases.^15,16^ As reported, hyperlipidemia could skew the transcriptional regulation of pro-inflammatory factors of myeloid cell, including mast cells, to promote myeloid cell extravasation and atherosclerosis.^17,18^ However, little is known about their involvement in cardiac fibrosis in hyperlipidemia mice.

In this study, we aimed to investigate whether hyperlipidemia is associated with cardiac fibrosis. Furthermore, we evaluated the activity of TGF-β/Wnt/β-catenin pathway, the expression levels of tryptase, chymase and α-SMA in hyperlipidemia mice.

## Methods

### Declaration of ethics in animals

Eight-week-old C57BL / 6 mice were purchased from the Experimental Animal Center of Dalian Medical University (Dalian, China). Mice were allowed access to water and food ad libitum, but fasted overnight with water available before surgery. All animal experiments were approved by the ethics committee of Dalian Medical University and performed in accordance with the institutional guidelines, and in accordance with the Helsinki Declaration.

### Induction of experimental hyperlipidemia

Via random number table, the animals were divided into two experimental groups (n = 8, for convenience) : control group received a normal diet , and the hyperlipidemia (H-lipid) group received high fat diet (HFD, D12451, Research diet, USA) for 8 weeks. Hyperlipidemia was documented by estimating the level of total cholesterol (TC) and triglycerides (TG) in serum using commercially available kits (*Jiancheng Biotech*, Nanjing, China).

### Morphological changes

After 8 weeks' high-fat diet, the mice were sacrificed. The hearts of mice were removed, and fixed in 10% (v/v) neutral formalin and processed by standard histological techniques. The hearts were stained with haematoxylin and eosin (H&E) and picrosirius red staining. Then they were examined for the morphological changes. The hearts of mice were also used to determinate expression of protein of chymase, tryptase, TGF-β, β-catenin and α-SMA by western blot analysis and immunohistochemical staining.

### Western blot analysis

According to the manufacturer's instructions, proteins were extracted from mice hearts with protein extraction kit (*KeyGen Biotech*, Nanjing, China). Protein was measured according to the procedure of bicinchoninic acid (BCA) (Solarbio, Beijing, China), with bovine serum albumin as the standard. Proteins (20 µg) were resuspended in electrophoresis sample buffer containing β-mercaptoethanol and separated by electrophoresis on a pre-cast 10% SDS-polyacrylamide gel (Bio-Rad, Hercules, CA), followed by electrotransfer to a PVDF membrane (Millipore, Bedford, MA). Membranes were blocked using 5% non-fat milk in Tris-buffered saline with 0.1% Tween-20 (TBST) for 2 h at 37°C. β-actin served as loading control. Membranes were incubated overnight at 4°C with a 1:1000 dilution of polyclonal antibody for tryptase, chymase, TGF-β, β-catenin and α-SMA respectively (*Beijing Boisynthesis Biotechnology*, China), and with a 1:1500 dilution of monoclonal antibody for β-actin (Beyotime, China). After subsequent washing with TBST, the blots were then incubated with secondary antibodies. After extensive washing with TBST, membranes were exposed to the enhanced chemiluminescence-plus reagents (ECL) from Beyotime Institute of Biotechnology (Haimen, China) according to the manufacturer's protocol. Emitted light was documented with a BioSpectrum-410 multispectral imaging system with a Chemi HR camera 410(Bio-Rad, Hercules, CA, USA). Protein bands were visualized and photographed under transmitted ultraviolet light. The image was used for semiquantitative measurements based on band densitometry.

### Immunohistochemical staining

Histological sections of mice hearts (4 µm thick) were mounted on poly-L-lysine-coated slides. Slides were deparaffinized in xylene and rehydrated in graded alcohols. Sections were pretreated with citrate buffer (0.01 mol/L citric acid, pH 6.0) for 20 min at 95 °C. Then, at room temperature, they were immersed in PBS containing 3% H_2_O_2_ for 10 min. After exposing them to 10% normal goat serum in PBS for 30 min at room temperature, the tissue sections were incubated at 4°C overnight with rabbit polyclonal anti-α-SMA and TGF-β (dilution 1:100). Then sections were rinsed with PBS, incubated with biotinylated goat anti-rabbit IgG for 20 min at room temperature and treated with 3,30-diaminobenzidine chromogen for 5 min at room temperature. Finally, sections were counterstained with hematoxylin for 6 min.

### Collagen quantification

Picrosirius red staining was performed with serially sectioned tissues. Paraffin-embedded tissues were deparaffinized in xylene, rehydrated in graded alcohols and then incubated in 0.1% Sirius Red solution for 1 h at room temperature. Finally, sections were counterstained with hematoxylin for 2 min. The sections were studied under a light microscope at different magnifications

### Data analysis

Statistical analysis was computed using SPSS 13.0 software. Group data were expressed as mean ± S.D. Shapiro-Wilk test was used to check the normality of the studied data, and, then, parametric or non-parametric tests were used for the analysis of normal or non-normal data distribution, respectively. Data with normal distribution underwent unpaired Student's sample t-test. In all statistical analyses, Two-sided p < 0.05 was considered to indicate a statistically significant result.

## Results

### Serum biochemistry changed after high-fat diet treatment

Following continuous feeding with high-fat diet for 8 weeks, serum levels of TC and TG in the H-lipid group were significantly higher than the control group (Table 1).

**Table 1:** Serum levels of TC and TG after high-fat diet for 8 weeks. (mmol/l)

Group	TG	TC
Control	0.73 ± 0.12	1.87 ± 0.25
H-lipid	1.21 ± 0.13	3.34 ± 0.33
p value	< 0.05	< 0.05

TC: total cholesterol; TG: triglycerides.

### Histological examinations

**Figure 1:**
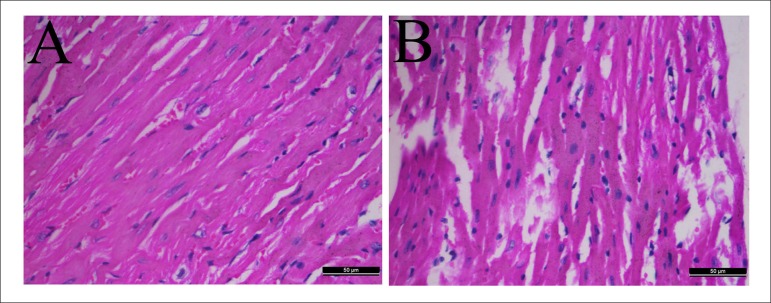
Change in heart photomicrographs of hyperlipidemic mice. Hearts were stained with H&E (A-B). Representative sections from hearts of a control mouse (A), hyperlipidemic mouse (B). H&E, magnification × 400.

H&E staining of heart tissues showed that myocardial fibers in control group were in order and their structure was normal. There was no broken fiber, the nucleus of myocardial cell was regular. However, the muscle fiber was arranged in disorder, extensively collapsed and degenerated in hyperlipidemia group (Figure 1).

### Hyperlipidemia increased mast cell chymase and tryptase production

**Figure 2:**
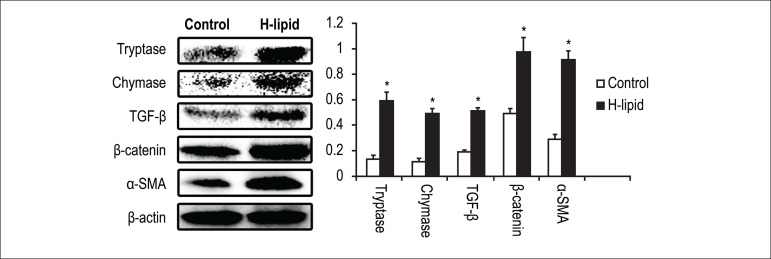
Effect of hyperlipidemia on the protein expression of tryptase, chymase, TGF-β, β-catenin and α-SMA in hyperlipidemic mice's hearts. The bar graph shows the relative expression ratio of each protein calculated after normalization by β-actin. Data are expressed as mean± S.D. (*p < 0.05 vs Control group; n = 8)

Since mast cell protease play essential role in fibrosis process, their production levels in the case of hyperlipidemia would be our concern. To investigate this, mice heart were immediately removed after sacrifice for western blot analysis. The results of western blot showed that the protein expressions of chymase and tryptase were increased significantly compared with the control group (Figure 2)

### Hyperlipidemia promoted activity of TGF-β and WNT/β-catenin pathway.

TGF-β is a pro-fibrotic cytokine that induces proliferation of macrophages and fibroblast through the induction of other growth factors. The Wnt/β-catenin signaling pathway is essential for the fibrosis induced by TGF-β. Western blot analysis of TGF-β and β-catenin showed that increased expression of both proteins were evident in H-lipid group compared to the control group. (Figure 2)

**Figure 3:**
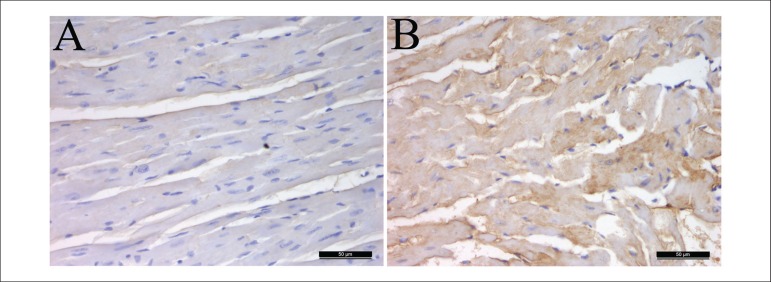
Effect of hyperlipidemia on the protein expression of TGF-β in hyperlipidemic mice's hearts. The hearts were immunohistochemical stained (A-B). Representative sections from hearts of a control mouse (A), a hyperlipidemic mouse (B). Immunohistochemical staining, magnification × 400.

The immunohistochemistry results were similar to the mentioned above. The protein expressions of TGF-β in the mice's hearts of H-lipid group were significantly increased compared with the control group (Figure 3).

### Hyperlipidemia induced great collagen accumulation in the heart

**Figure 4:**
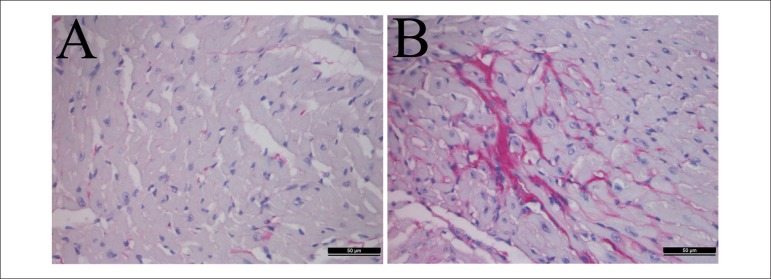
Effect of hyperlipidemia on the collagen accumulation in hyperlipidemic mice's hearts. The hearts were picro-sirius red stained (A-B). Representative sections from hearts of a control mouse (A), hyperlipidemic mouse (B). Picro-sirius red staining, magnification ×400.

**Figure 5:**
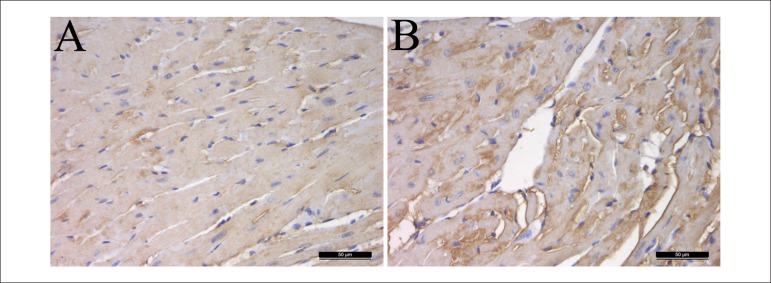
Effect of hyperlipidemia on the protein expression of α-SMA in hyperlipidemic mice's hearts. The hearts were immunohistochemical stained (A-C). Representative sections from hearts of a control mouse (A), hyperlipidemic mouse (B). Immunohistochemical staining, magnification × 400.

Picrosirius red staining suggested significant greater collagen content in the H-lipid group compared with the control groups (Figure 4) moreover, the immunohistochemistry (Figure 5) and western blot (Figure 2) results showed that the expression levels of α-SMA in the heart tissues of the H-lipid group were increased significantly compared with the control group.

## Discussion

Hyperlipidemia is a clinical and metabolic disorder characterized by abnormal elevation of the major circulatory lipid and lipoprotein levels that accounts for approximately 56% cases of cardiovascular diseases worldwide and causes about 4.4 million deaths annually.^19^ As reported, the death risk from ischemic heart disease is significantly high in patients with essential hyperlipidemia.^20^

In addition, it was reported that high-calorie diet feeding could induce hyperlipidemia and promoted heart injury and failure in rats.^21^ However, there is little knowledge about the mechanisms underlying these biological changes. Cardiac fibrosis is a common pathologic feature of end-stage heart disease. Therefore, as potential causes of cardiac fibrosis have to be investigated, we researched whether hyperlipidemia is associated with the cardiac fibrosis progression, contributing to the pathogenesis of heart failure. In the present study, the level of TC and TG in serum was significantly higher in H-lipid group, which indicates that the model of hyperlipidemia mice has been established successfully. Then we investigated the changes in mice heart morphology by H&E staining. The results showed muscular layer of heart arranged in disorder with fracture in mice of the model group. Picrosirius red staining suggested significantly greater collagen content in the H-lipid group compared with the control groups, and western blot showed that the expression level of α-SMA, known as marker of myofibroblast, in H-lipid group was significantly higher than in control group.

Mast cells have been recognized as important effector cells in tissue fibrosis. As reported, mast cells express serine proteases; tryptase and chymase that are associated with fibrosis in various diseases. However, little is known about their involvement in heart disease induced by hyperlipidemia. In our study, the results of western blot showed that the protein expressions of tryptase and chymase in the mice's heart of H-lipid group were increased compared with the control group. To further explore the molecular mechanism of cardiac fibrosis in hyperlipidemia mice, using western blot analysis and immunohistochemical staining, we examined the protein expressions of TGF-β which are intricately linked with the profibrotic effects of mast cells, and the Wnt/β-catenin pathway that is essential for the fibrosis induced by TGF-β. The results of western blot analysis and immunohistochemical staining demonstrated that the protein expressions of TGF-β in the mice's heart of H-lipid group were significantly increased compared with the control group. Moreover, the results of western blot analysis showed that the changed protein expression level of β-catenin was similar to TGF-β.

## Conclusions

In summary, as the pathogenesis indicates, the progression of cardiac fibrosis may be induced by hyperlipidemia. Interestingly, heart tissues from hyperlipidemia mice revealed increased mast sell activation e upregulated activity of TGF-β/Wnt/β-catenin pathway. The results of this study demonstrated that the mast cells and TGF-β/Wnt/β-catenin pathway were not only very important for the cardiac tissue fibrosis in hyperlipidemia but also a possible target for therapy.
